# Gut–liver–muscle axis: linking gut microbiota dysbiosis to malnutrition and sarcopenia in liver disease

**DOI:** 10.3389/fmed.2025.1727270

**Published:** 2026-01-15

**Authors:** Hongjing Zhao, Yang Liu, Linhao Su, Panpan Cui, Jinhai Sai, Sijin Li, Na Wang, Peiyuan He

**Affiliations:** 1 Hebei Key Laboratory of Panvascular Diseases, Department of Gastroenterology and Hepatology, The Affiliated Hospital of Chengde Medical College, Hebei, China; 2 Department of Biomedical Engineering, Chengde Medical University, Hebei, China; 3 Hebei Key Laboratory of Gastroenterology, Department of Gastroenterology, Hebei Institute of Gastroenterology, The Second Hospital of Hebei Medical University, Hebei, China

**Keywords:** gut-liver-muscle axis, gut microbiota, liver disease, malnutrition, sarcopenia, short-chain fatty acids, probiotics, fecal microbiota transplantation

## Abstract

Nutritional disorders and muscle wasting associated with liver disease are key determinants of poor prognosis in patients with chronic liver disease. The formation of these conditions involves multiple factors, including impaired energy metabolism, enhanced protein degradation, and gut microbiota imbalance. In recent years, with the deepening of microbiome research, the concept of the “gut-liver-muscle axis” has gradually emerged to explain the more systematic interaction between gut microbiota, liver metabolism, and skeletal muscle homeostasis. Gut dysbiosis can promote liver inflammation and metabolic disorders through various pathways, further weakening muscle energy utilization and protein synthesis, ultimately leading to malnutrition and sarcopenia. This review systematically explores the crucial role of gut microbiota in liver disease-related malnutrition and muscle wasting, elucidates its potential mechanisms in influencing host metabolism and nutritional status through the “gut-liver-muscle axis,” and discusses the prospects of microbiome interventions in improving nutritional outcomes in liver disease.

## 1 Introduction

Liver disease is a major global public health burden, and its chronic progression is often accompanied by malnutrition, muscle wasting, and metabolic disorders (1). In the context of chronic liver disease, malnutrition is a clinical syndrome characterized by measurable adverse effects on body composition or physiological functions due to insufficient or excessive nutrient intake (2). Epidemiological studies show that approximately 20%–60% of cirrhosis patients experience varying degrees of malnutrition, which not only affects patients’ quality of life and treatment outcomes but also significantly increases the risks of infection, complications, fractures, disability, and mortality (3).

The research on the gut-liver axis in recent years has provided a new framework for understanding metabolism disorders related to liver disease. As a crucial organ for digestion, absorption, and immunity, the gut’s microbial composition and barrier integrity can be altered, allowing bacterial products to be directly transported to the liver via the portal vein, triggering inflammatory responses and further exacerbating energy and protein metabolism disorders (4). Meanwhile, bile acids produced by the liver can also impact the gut microbiota composition, demonstrating a bidirectional regulatory relationship (5). Building on this, recent studies have introduced the concept of the “gut-liver-muscle axis,” where gut microbiota influences liver energy supply and inflammation through pathways such as short-chain fatty acids, bile acids, and amino acid metabolism, thereby indirectly regulating skeletal muscle protein synthesis and degradation (6). This framework provides a new biological foundation for understanding liver disease-related malnutrition and sarcopenia.

Therefore, systematically reviewing the mechanisms by which gut microbiota contributes to liver disease-related malnutrition and sarcopenia not only helps to reveal the metabolic roots of disease development but also provides new possibilities for nutritional interventions and microbiome-based therapies. This paper will review the relevant research progress in three areas: gut microbiota and liver disease-related malnutrition, the gut-liver-muscle axis, and microbiome interventions, and will offer perspectives on future research directions.

## 2 Liver disease-related malnutrition

Liver disease-related malnutrition refers to an imbalance in nutrient supply caused by insufficient energy intake, absorption barriers, or impaired metabolic function in the context of chronic liver disease, especially cirrhosis. This imbalance leads to changes in body composition, weakness, and functional decline (7). Its onset and progression involve multiple mechanisms, including energy metabolism disorders, inflammatory responses, hormonal regulation abnormalities, and micronutrient imbalances (8). It is important to emphasize that malnutrition does not solely refer to weight loss but is a spectrum of nutritional disorders across the entire body mass index (BMI) range—from underweight to obesity. In patients with cirrhosis, it is commonly manifested as changes in body composition, weakness, and sarcopenia. The phenotypes of malnutrition can be categorized into two types: nutrient deficiency and nutrient excess (9). Nutrient deficiency is characterized by protein-energy malnutrition (PEM), usually accompanied by weight loss, muscle wasting, and decreased physical strength (10, 11). On the other hand, nutrient excess is commonly seen in sarcopenic obesity, where patients accumulate fat but experience muscle loss (12, 13). Liver disease-related malnutrition not only affects the patient’s nutritional status but also exacerbates weakness, making patients more prone to complications and reducing their quality of life.

Sarcopenia is one of the key phenotypes of liver disease-related malnutrition. According to the definition by the European Working Group on Sarcopenia, sarcopenia is a progressive, systemic skeletal muscle disease characterized by a decrease in muscle mass, accompanied by a decline in muscle strength or function. In cirrhosis research, sarcopenia is typically regarded as the primary phenotype of reduced skeletal muscle mass and is an important indicator of prognosis in patients with chronic liver disease (14). The development of sarcopenia is closely related to chronic inflammation, metabolic disorders, insulin resistance, and other factors (15). Recent studies have shown that gut dysbiosis plays a key role in the onset of liver disease-related malnutrition, as it contributes to the formation of malnutrition and sarcopenia by affecting metabolite production, immune response, and inflammation levels, among other pathways (16). Overall, the interrelationship between liver disease-related malnutrition and sarcopenia requires systematic evaluation and comprehensive interventions to improve the clinical prognosis of patients.

## 3 Gut-liver-Muscle axis

Recent research into gut microbiota and liver disease metabolism has led to the emergence of the “gut-liver-muscle axis,” an integrated framework that explains the multi-organ interactions in malnutrition and sarcopenia in chronic liver disease (17, 18). This concept posits that the gut, liver, and skeletal muscles interact through multiple pathways, including microbial-derived metabolites, nutrient substrate supply, inflammatory signals, and hormonal regulation, collectively contributing to the maintenance of whole-body energy and protein metabolism homeostasis.

The formation of this theory is based on the gradual accumulation of evidence from multiple perspectives. Early studies revealed the bidirectional associations between gut dysbiosis, liver metabolic disorders, and muscle wasting from the viewpoints of the gut-liver axis and liver-muscle axis (19, 20). Based on these, research gradually placed these three components within the same physiological network, proposing a continuous relationship between changes in the gut microbiota, liver metabolic state, and muscle function decline, thus establishing the framework for the “gut-liver-muscle axis” concept. An increasing number of studies have shown that the gut microbiota, by modulating liver metabolism and immune responses, directly or indirectly influences muscle energy metabolism and protein synthesis. For example, short-chain fatty acids, products of gut bacteria, can enhance muscle cell energy supply through their impact on liver metabolism, while also alleviating muscle wasting (21). These studies provide important foundational data for a deeper understanding of the “gut-liver-muscle axis” and promote multidimensional exploration of the relationship between gut microbiota, liver metabolism, and muscle health.

Although this theoretical model has been preliminarily established, direct research focusing on the overall interactions between the three components remains limited, with most studies still focusing on two-organ level analyses. Therefore, systematically reviewing the intrinsic connections within the gut-liver-muscle axis will not only deepen our understanding of the mechanisms behind chronic liver disease-related malnutrition and sarcopenia but also offer new research directions for exploring microbiome interventions, metabolic regulation, and integrated nutritional strategies.

## 4 Gut microbiota and liver disease-related malnutrition

Chronic liver disease is commonly accompanied by significant gut dysbiosis, characterized primarily by a decrease in microbial diversity, a reduction in beneficial bacteria, and an enrichment of opportunistic pathogens (22). These microbial changes can impair bile acid circulation, short-chain fatty acid (SCFA) production, nitrogen metabolism, and gut barrier integrity, leading to lipid and protein-energy metabolic disorders and maintaining a chronic inflammatory state (23, 24). Liver diseases of different etiologies exhibit distinct patterns of dysbiosis and can affect host metabolism and inflammatory responses through various pathways. Shared and disease-specific microbiota-mediated mechanisms contributing to malnutrition across liver diseases are summarized in Table 1.

**TABLE 1:** Gut microbiota alterations, shared and disease-specific mechanisms linking liver diseases to malnutrition and muscle loss.

Liver disease type	Characteristic gut microbiota alterations	Shared microbiota-related mechanisms	Disease-specific key mechanisms	Nutritional and body composition outcomes
Non-alcoholic fatty liver disease (NAFLD)	Increased Firmicutes and reduced Bacteroidetes; enrichment of Proteobacteria; depletion of SCFA-producing taxa (e.g.,Faecalibacterium, Roseburia)	Gut barrier impairment; increased LPS translocation; reduced SCFA production; chronic low-grade inflammation	Impaired FXR/TGR5 signaling; SREBP-1c–driven lipogenesis; insulin resistance	Reduced energy utilization efficiency; fat accumulation with muscle mass decline; sarcopenic obesity and increased risk of malnutrition
Alcoholic liver disease (ALD)	Reduced microbial diversity; enrichment of Proteobacteria and Enterobacteriaceae; depletion of Lactobacillus and Bifidobacterium	Intestinal barrier disruption; endotoxemia; SCFA (especially butyrate) deficiency; sustained inflammation	Alcohol- and acetaldehyde-induced mitochondrial dysfunction; impaired fatty acid oxidation; increased ammonia production and urease activity	Protein–energy malnutrition; accelerated muscle wasting; increased risk of sarcopenia
Viral hepatitis (HBV/HCV)	Reduced microbial diversity; depletion of SCFA-producing bacteria; enrichment of opportunistic pathogens (e.g., Enterococcus, Escherichia–Shigella)	Increased intestinal permeability; persistent LPS translocation; chronic inflammation; insufficient energy substrates	Virus-driven immune activation; elevated basal energy expenditure; unresolved inflammatory responses	Appetite suppression; enhanced protein catabolism; progressive loss of muscle mass
Autoimmune liver diseases (AIH/PBC/PSC)	Marked reduction of Bifidobacterium, Faecalibacterium, Roseburia, and Lachnospiraceae; relative enrichment of opportunistic taxa	Reduced microbial metabolites; impaired gut barrier stability; enhanced inflammatory signaling	Th17/Treg imbalance; impaired immune tolerance; potential molecular mimicry–mediated immune activation	Increased inflammation-related energy expenditure; accelerated muscle protein breakdown; elevated malnutrition risk
Liver Cirrhosis	Severe dysbiosis; enrichment of Enterobacteriaceae, Streptococcus, and Veillonella; invasion of oral-origin bacteria; depletion of beneficial taxa	Profound gut barrier failure; increased endotoxin and bacterial metabolite burden; sustained inflammation	Disordered ammonia metabolism (increased ammonia-producing bacteria and urease activity); impaired hepatic detoxification	Severe malnutrition; high prevalence of sarcopenia; poor clinical prognosis

### 4.1 Non-alcoholic fatty liver disease (NAFLD)

Non-alcoholic fatty liver disease (NAFLD) is a liver lipid deposition disease that occurs in the context of insulin resistance and metabolic syndrome, and its development is closely associated with gut dysbiosis (25). Studies have shown that NAFLD patients typically exhibit microbiome characteristics such as reduced SCFA-producing bacteria, increased Proteobacteria, and altered Firmicutes/Bacteroidetes ratios (26, 27). These microbiome changes not only affect local gut metabolic functions but also, by regulating bile acid composition, energy substrate supply, and inflammation levels, exert systemic effects on liver metabolism and contribute to the development of NAFLD-related malnutrition (28–31).

At the metabolic level, gut dysbiosis leads to elevated lipopolysaccharide (LPS) levels, which cause glucose and lipid metabolism dysfunction, thereby exacerbating NAFLD (32). Moreover, the bile acid conversion abnormalities caused by gut dysbiosis can impair FXR/TGR5-mediated liver fatty acid oxidation and energy metabolism regulation, leading to the overactivation of SREBP-1c-related lipogenesis pathways, further aggravating hepatic lipid accumulation (33–35). In addition, insufficient production of short-chain fatty acids (SCFAs), especially butyrate, limits energy supply to the intestinal epithelium, decreases tight junction protein expression, and weakens the gut barrier (36). Gut-derived substances like LPS more easily enter the liver, triggering mild persistent inflammation and exacerbating mitochondrial dysfunction. This “microinflammation-mitochondrial damage” pattern is considered a major cause of decreased energy utilization efficiency in NAFLD patients (37–40).

Insulin resistance is one of the pathological features of NAFLD, and gut microbiota influences systemic insulin sensitivity by modulating SCFA levels and their effects on gut hormones (GLP-1, PYY), further reducing insulin sensitivity (41–43). When muscle response to insulin decreases, protein breakdown tends to increase, and even with normal overall nutrient intake, an “insufficient energy utilization, limited protein synthesis” state can develop. This metabolic pattern explains why NAFLD patients with an obesity phenotype may still experience muscle loss, abnormal body composition, and “malnutrition manifestations” (44).

Furthermore, the barrier disruption and increased inflammation caused by gut dysbiosis further deplete energy and impair liver metabolic function, leading to a “supply-demand imbalance” when the body faces nutritional and metabolic demands (45, 46). Therefore, changes in the gut microbiota not only participate in the onset and progression of NAFLD but may also drive the development of nutrient excess-related malnutrition in NAFLD patients, increasing the risk of muscle wasting and sarcopenia through their impact on lipid metabolism, insulin sensitivity, inflammation levels, and energy utilization efficiency (47, 48).

### 4.2 Alcoholic liver disease (ALD)

Alcoholic liver disease (ALD) is caused by long-term excessive alcohol consumption, and its progression can manifest in stages such as steatosis, alcoholic hepatitis, and liver fibrosis (49). Studies have shown that changes in the composition and function of the gut microbiota may be associated with the onset and progression of ALD (50). Chronic alcohol consumption is often accompanied by a decrease in microbial diversity, along with an increase in the relative abundance of bacteria such as Proteobacteria and Enterobacteriaceae, while beneficial bacteria like Lactobacillus and Bifidobacterium tend to decrease (51–53). This dysbiosis disrupts gut barrier function, leading to a reduction in tight junction protein expression, allowing gut-derived substances to enter the portal vein, thereby activating inflammatory pathways in the liver (54, 55).

In addition to the inflammatory response, gut dysbiosis and alcohol metabolism jointly affect the host’s nutritional metabolism. First, barrier disruption and gut inflammation can reduce the abundance of SCFA-producing bacteria, leading to insufficient supply of key energy substrates like butyrate, further weakening the energy metabolism capacity and protective effects of the gut mucosa, which in turn indirectly exacerbates the metabolic burden on the liver (56–58). Secondly, alcohol metabolites such as acetaldehyde and alcohol-induced oxidative stress can damage mitochondrial function, and in combination with bile acid metabolism dysregulation, this reduces fatty acid oxidation efficiency and weakens energy utilization capacity, driving protein-energy metabolism disorders (59, 60). Additionally, some studies suggest that the reduced expression of Apolipoprotein H (ApoH) may contribute to dysregulated metabolic pathways, further affecting lipid and energy metabolism and promoting hepatic lipid deposition (61).

Alcohol-related changes in the gut microbiota, through their impact on inflammation, energy metabolism, and bile acid metabolism, may exacerbate malnutrition and muscle wasting in ALD patients, thereby increasing the risk of sarcopenia.

### 4.3 Other non-metabolic liver diseases

In addition to metabolic liver diseases, patients with viral hepatitis and autoimmune liver diseases often exhibit reduced gut microbiota diversity, decreased SCFA-producing bacteria, and an increase in the relative abundance of opportunistic pathogens (62–65). These changes may affect gut barrier stability, making gut-derived substances more likely to enter the portal vein system and be associated with enhanced hepatic immune activation and inflammatory responses (66). At the same time, the reduction in SCFA-producing bacteria and their impact on energy substrate supply and immune regulation may make patients more susceptible to prolonged inflammation and metabolic depletion, thereby increasing the risk of malnutrition and muscle wasting (67, 68).

In viral hepatitis, dysbiosis is often linked to the maintenance of chronic inflammation and impaired energy metabolism. Persistent infection with Hepatitis B Virus (HBV) or Hepatitis C Virus (HCV) may be accompanied by a decrease in SCFA-producing bacteria and an increase in pathogenic bacteria, which weakens the gut barrier and allows substances like LPS to enter the liver, thus making inflammation difficult to resolve (69, 70). Chronic inflammation may suppress appetite, promote protein breakdown, and increase basal metabolic rate, making patients more likely to experience nutritional decline and muscle loss (71).

In autoimmune liver diseases, gut dysbiosis is more prominently manifested as immune regulatory dysfunction. Accumulating evidence indicates that alterations in gut microbial composition are closely associated with a reduction in regulatory T cells, imbalance of the Th17/Treg axis, and impaired immune tolerance (72). Under conditions of disrupted immune homeostasis, gut-derived microbial antigens and their associated components are more likely to participate in antigen presentation processes, and certain microorganisms may activate T-cell responses through molecular mimicry, thereby sustaining or amplifying hepatic autoimmune reactions (73). Meanwhile, impairment of the intestinal barrier facilitates the persistent translocation of microbe-associated molecular patterns into the portal circulation, resulting in chronic, low-grade but sustained immune stimulation that maintains the liver in a state of chronic inflammatory activation (74). In this pro-inflammatory milieu, continuous release of inflammatory mediators can increase basal metabolic rate, promote protein catabolism, and inhibit skeletal muscle protein synthesis, thereby predisposing patients to malnutrition and muscle mass loss and increasing the risk of sarcopenia (75).

Overall, while dysbiosis in viral hepatitis and autoimmune liver diseases shares some common features with metabolic liver diseases, it exhibits more pronounced disease-specificity in terms of persistent inflammation, immune regulation abnormalities, and impaired energy metabolism. These microbiome and immune-metabolic changes together form the potential biological basis for nutritional decline and muscle wasting in such chronic liver diseases.

### 4.4 Liver cirrhosis

Liver cirrhosis is the end stage of various chronic liver diseases, characterized by the progression of liver fibrosis, impaired liver function, and systemic metabolic abnormalities (76). Studies show that cirrhosis patients commonly exhibit significant dysbiosis in their gut microbiota, including reduced diversity, decreased beneficial bacteria, and an increase in the relative abundance of opportunistic pathogens and oropharyngeal bacteria (77–80). These changes are especially prominent during the decompensated phase and are associated with poor clinical prognosis (81, 82).

Liver dysfunction affects bile acid secretion and gut barrier stability, making the gut environment more susceptible to colonization by opportunistic pathogens (83). Weakened gut barriers may exacerbate the translocation of gut-derived substances, which is associated with persistent liver inflammation and fibrosis progression (84).

Abnormal ammonia metabolism is one of the metabolic features in cirrhosis. Dysbiosis may lead to an increase in ammonia-producing bacteria and urease activity, while the liver’s ability to clear ammonia decreases, resulting in elevated blood ammonia levels (85). High ammonia levels not only affect central nervous system function but also may inhibit muscle protein synthesis and impair energy metabolism, making it a crucial mechanism for cirrhosis-related sarcopenia (86, 87). Some studies suggest that improving gut barrier integrity or adjusting microbiota composition may help reduce blood ammonia levels, improve metabolic status, and slow disease progression (88, 89).

Overall, dysbiosis in cirrhosis is more prominently characterized by barrier disruption and ammonia metabolism abnormalities, which may increase the risk of malnutrition and muscle wasting by affecting energy and protein metabolism.

## 5 Gut microbiota and liver disease-related sarcopenia

**FIGURE 1:**
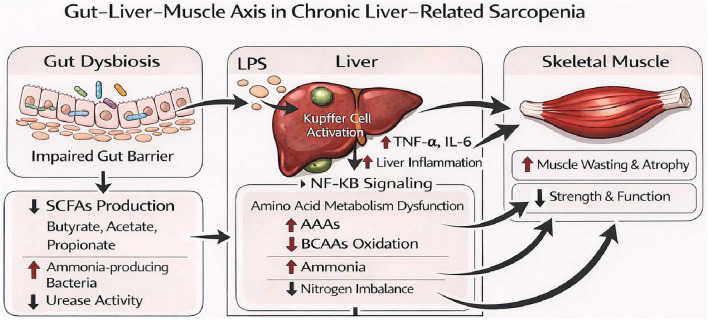
Gut–liver–muscle axis in chronic liver disease–related sarcopenia. This figure illustrates the gut–liver–muscle axis involved in sarcopenia associated with chronic liver disease. Gut dysbiosis, characterized by impaired intestinal barrier function and reduced short-chain fatty acid (SCFA) production, promotes bacterial translocation and lipopolysaccharide (LPS) release. LPS activates Kupffer cells, inducing pro-inflammatory cytokines such as tumor necrosis factor-α (TNF-α) and interleukin-6 (IL-6) and sustaining hepatic inflammation through NF-κB signaling. Persistent inflammation leads to amino acid metabolic disturbances, including impaired branched-chain amino acid (BCAA) utilization, increased aromatic amino acids (AAAs), and nitrogen imbalance, thereby promoting skeletal muscle protein catabolism and inhibiting protein synthesis. Reduced SCFA production may further aggravate skeletal muscle metabolic dysfunction by disrupting nitrogen homeostasis and nutrient substrate utilization. In parallel, increased ammonia-producing bacteria exacerbate hyperammonemia, impair muscle energy metabolism, and accelerate muscle wasting, ultimately resulting in reduced muscle mass, strength, and function.

Liver disease–related sarcopenia is a key component of malnutrition in chronic liver disease, characterized by progressive declines in skeletal muscle mass and strength (90). Clinical studies have shown that patients with cirrhosis and sarcopenia exhibit more profound gut dysbiosis, with significantly reduced microbial diversity compared with those without sarcopenia and healthy individuals (91, 92). Specifically, decreases in short-chain fatty acid–producing bacteria, such as Faecalibacterium and Roseburia, alongside enrichment of Enterococcus, ammonia-producing bacteria, and oropharyngeal taxa have been observed. These microbial alterations are closely associated with hyperammonemia, heightened systemic inflammation, and accelerated muscle loss (93, 94). Accumulating evidence suggests that gut dysbiosis is not merely an epiphenomenon but actively contributes to the development and progression of sarcopenia through interconnected mechanisms involving inflammatory signaling, nitrogen metabolism imbalance, and energy metabolic dysfunction, as schematically illustrated in Figure 1 (95, 96).

Firstly, gut barrier disruption and bacterial translocation allow LPS to enter the liver via the portal vein, activating Kupffer cells and inducing the elevation of pro-inflammatory cytokines such as TNF-α and IL-6 (97). These inflammatory cytokines not only exacerbate liver damage but also promote skeletal muscle protein degradation and inhibit protein synthesis by activating the NF-κB signaling pathway, accelerating muscle atrophy (98, 99). Studies have shown that inhibiting gut-derived LPS-induced inflammatory signaling can significantly alleviate LPS-related muscle protein degradation and atrophy phenotypes, suggesting that blocking LPS-mediated inflammatory pathways is a potential strategy to improve inflammation-related muscle wasting (100).

Secondly, amino acid metabolism abnormalities are an important mechanism of liver disease-related sarcopenia (101). Liver dysfunction caused by gut dysbiosis leads to amino acid metabolism imbalances, with limited oxidation of branched-chain amino acids (BCAAs) and accumulation of aromatic amino acids (AAAs), resulting in nitrogen metabolism imbalance (102, 103). This nitrogen metabolism imbalance not only affects liver metabolic function but also impairs muscle protein synthesis and degradation, leading to skeletal muscle atrophy and muscle wasting. Specifically, nitrogen metabolism disorders exacerbate skeletal muscle energy metabolism dysfunction and inhibition of protein synthesis, promoting muscle loss and functional decline. At the same time, dysbiosis, which leads to an increase in ammonia-producing bacteria and elevated urease activity, further exacerbates hyperammonemia, suppressing muscle energy metabolism and protein synthesis (104–106). Previous studies have shown that regulating gut microbiota or supplementing BCAAs can improve nitrogen metabolism status and enhance muscle mass. BCAAs have been shown to effectively improve hypoalbuminemia in cirrhosis patients, prevent sarcopenia, and reduce skeletal muscle fat accumulation by promoting the mTOR signaling pathway, enhancing muscle protein synthesis, and inhibiting muscle degradation. The gut microbiota regulates skeletal muscle energy metabolism and protein synthesis through its effects on immune responses and metabolic products (107–109).

Additionally, SCFAs, as important metabolic products of the gut microbiota, play a crucial role in providing energy to skeletal muscles (110). Butyrate can activate AMPK and enhance mitochondrial oxidative capacity (111), acetate and propionate are involved in gluconeogenesis and fatty acid metabolism (112, 113). A reduction in SCFA production due to gut dysbiosis weakens these protective effects (114). Supplementing with butyrate salts or probiotics that promote SCFA production not only improves liver lipid metabolism and inflammation but also enhances skeletal muscle mitochondrial function, glycogen storage, and muscle strength (115, 116). This suggests that SCFAs play a key regulatory role in energy metabolism disorders and sarcopenia. Furthermore, regulating bile acid metabolism can significantly improve NAFLD induced by high-fat diets, reducing hepatic lipid accumulation and improving liver function, supporting the connection between bile acid signaling dysregulation and lipid metabolism disorders (117).

In summary, gut dysbiosis contributes to the development of liver disease-related sarcopenia through multiple pathways, including inflammation, amino acid metabolism, and energy homeostasis. Further elucidation of the molecular mechanisms of the “gut-liver-muscle axis” combined with microbiome-based interventions may provide new therapeutic directions for improving muscle mass and overall nutritional status in chronic liver disease patients.

## 6 Microbiome interventions and nutritional therapy

Given the important role of gut microbiota in liver disease-related metabolic disorders and sarcopenia, various microbiome and nutritional interventions have been used in recent years to improve the function of the gut-liver-muscle axis. Studies indicate that these interventions not only modulate gut microbiota composition and liver metabolism but also promote muscle protein synthesis by improving inflammation, energy supply, and amino acid utilization, thereby alleviating liver disease–related malnutrition and sarcopenia. The effects of microbiota-targeted interventions on liver–muscle metabolic outcomes are summarized in Table 2.

**TABLE 2:** Interventions for liver disease-related sarcopenia: mechanisms, effects, and clinical significance.

Intervention	Mechanism of action	Effects and clinical significance	Relevant literature
Probiotics	Improve gut microbiota structure, enhance gut barrier function, reduce gut-derived inflammation, increase SCFA production, regulate bile acid-FXR/TGR5 signaling	Improve liver metabolism, reduce low-grade inflammation, provide a favorable metabolic environment for muscle protein synthesis, promote muscle function recovery	Prokopidis et al. (118); Toda et al. (119); Rong et al. (120)
Synbiotics	Increase SCFA levels, modulate bile acid signaling, reduce oxidative stress in liver and muscle metabolism	Restore microbiota balance, improve muscle synthesis and function, potential to alleviate liver disease-related sarcopenia	Roychowdhury et al. (122); Asghari et al. (123)
Non-living bacterial products	Regulate gut microbiota, improve bile acid-serotonin signaling, reduce gut-derived inflammation, improve NAFLD metabolic disturbances	Indirectly improve muscle synthesis and energy utilization, potential therapeutic effect on liver disease-related sarcopenia	Tsuchiya et al. (124); Wang et al. (125); Ding et al. (126)
Fecal microbiota transplantation (FMT)	Reconstruct gut microbiota balance, increase SCFA-producing bacteria, reduce Gram-negative bacteria, improve blood ammonia levels, modulate bile acid and immune signaling	Restore gut-liver axis function, improve energy supply and inflammation, support muscle protein synthesis and function recovery	Ichim et al. (127); Bajaj et al. (128); Madsen et al. (129)
Nutritional support and integrated interventions	Supplement BCAAs, dietary fiber, and PUFAs, promote SCFA production, improve nitrogen balance, regulate microbiota, reduce inflammation, improve muscle energy metabolism	Improve nitrogen metabolism and muscle function, alleviate hepatic encephalopathy and sarcopenia, integrated intervention enhances gut-liver-muscle axis function	Singh Tejavath et al. (132); Li et al. (133); Sato et al. (134)

### 6.1 Probiotics and related microbiome interventions

Probiotics, prebiotics, and synbiotics are common strategies for regulating gut microbiota. They can influence the gut-liver-muscle axis by improving gut microbiome structure, enhancing barrier function, and reducing gut-derived inflammation (118). Probiotic interventions containing Lactobacillus and Bifidobacterium have been reported to reduce serum LPS, improve insulin sensitivity, and alleviate low-grade inflammation, which may provide a more favorable metabolic environment for muscle protein synthesis (119). Prebiotics such as inulin and oligofructose promote SCFA production, enhance mucosal energy supply, and regulate appetite and glucose metabolism, which are thought to improve nutritional status and muscle function (120, 121).

Synbiotics, as compared to single-agent formulations, are more effective in promoting the restoration of microbiome homeostasis and may affect liver and muscle metabolism by increasing SCFA levels, regulating bile acid-FXR/TGR5 signaling, and reducing oxidative stress (122). Furthermore, “next-generation probiotics” like Akkermansia muciniphila have shown potential in animal studies to improve glucose utilization, enhance energy metabolism, and alleviate fatty liver. These changes may also benefit muscle health by improving the systemic metabolic environment (123).

Non-living bacterial products such as lactoferrin are thought to improve NAFLD-associated metabolic disorders by regulating gut microbiota, reducing gut-derived inflammation, and improving bile acid-serotonin signaling (124–126). These effects may indirectly improve muscle synthesis metabolism and energy utilization, offering potential significance for liver disease-related sarcopenia.

Overall, microbiome interventions provide a new therapeutic direction for restoring gut-liver-muscle axis function by reducing inflammation and improving energy and amino acid metabolism.

### 6.2 Fecal microbiota transplantation (FMT)

Fecal microbiota transplantation (FMT), by reconstructing gut microbiota balance, can improve the microbiome structure in cirrhosis and metabolic liver diseases (127, 128). In cirrhosis patients, FMT has been reported to increase SCFA-producing bacteria, reduce the proportion of Gram-negative bacteria, and decrease endotoxin levels. These changes not only help restore gut-liver axis function but also may improve energy supply and inflammation, providing a more stable metabolic environment for muscle protein synthesis.

In hepatic encephalopathy, FMT can reduce ammonia-producing bacteria and urease activity, improve blood ammonia levels, and enhance cognitive function (129, 130). The reduction in blood ammonia not only improves central metabolism but also alleviates the detoxification pressure of ammonia on skeletal muscles, helping to maintain muscle mass. Some studies also suggest that FMT can regulate bile acids and immune signaling, improving lipid metabolism and inflammation in NAFLD/NASH (131), potentially offering indirect benefits for muscle metabolism.

In the future, combining FMT with nutritional support may further enhance the metabolic recovery of the gut-liver-muscle axis.

### 6.3 Nutritional support and integrated interventions

Nutritional support remains an important component of liver disease management, and its impact on the gut-liver-muscle axis has also garnered attention. Studies have shown that moderate supplementation with BCAAs can promote muscle protein synthesis, improve nitrogen balance, and has been confirmed to help improve hepatic encephalopathy and sarcopenia (132). Dietary fibers and polyunsaturated fatty acids (PUFAs) can regulate microbiota composition, promote SCFA production, reduce inflammation, and may improve muscle energy metabolism (133, 134).

The future trend is to combine nutritional therapy with microbiome interventions to form an integrated “microbiota-metabolism-muscle” treatment strategy. By simultaneously improving gut microbiota, liver metabolism, and muscle synthesis capacity, this approach systematically enhances the function of the gut-liver-muscle axis, providing more precise nutritional management methods for liver disease patients.

## 7 Perspectives

With the development of multi-omics technologies and metabolic network analysis, the mechanisms by which gut microbiota contribute to liver disease-related malnutrition will be more deeply elucidated. Future research should focus on the following directions: establishing standardized microbiome assessment systems to identify microbiome characteristics at different stages of liver disease; exploring the signaling pathways of microbiome metabolites in nutritional metabolism regulation; developing personalized microbiome intervention strategies to achieve “precision nutrition” treatment; and combining artificial intelligence and metabolic modeling to construct dynamic predictive models of the gut-liver-muscle axis for early intervention and prognosis assessment.

In summary, the gut microbiota is not only a pathological participant in liver disease-related malnutrition but also a potential therapeutic target. By integrating microbiome regulation with nutritional therapy, systemic interventions from metabolic dysregulation to nutritional rehabilitation for liver disease patients may be achieved.
